# Answers to Querists

**Published:** 1867-09

**Authors:** 


Answers to Querists.—*Properties, Generation and Administration of Nitrous Oxide Gas.*

*Query 1st.*—Properties of Nitrous Oxide Gas? *Answer.* This gas is evolved by heat from the salt Nitrate of Ammonia, at a temperature of about 400° Fahr.

The salt first melts and then boils away, being dissolved entirely into vapor of water and into a permanent gas which can be collected over water in a gasometer. Nitrous oxide gas may also be obtained by dissolving zinc in dilute nitric acid. It is colorless, of a sweetish taste and a pleasant smell; its symbol being N O. and its specific gravity 1.527, and "every two volumes of the gas contain two volumes of nitrogen and one volume of oxygen, the whole being condensed or contracted one-third; a constitution resembling that of vapor of water." Eighty grains of nitrate of ammonia, will yield forty four grains of nitrous oxide, and thirty-six grains of water; cold water absorbs about its own volume of this gas. This fact explains the loss of gas, when the water in the gasometer is fresh; by using tepid water less gas is wasted. Sir Humphrey Davy in 1799 first discovered its anæsthetic property upon inhalation, and was also the first to apply it to dental purposes, as is shown in the following paragraph, which appears in the edition of his works edited by his brother Dr. John Davy, London, 1839. The third volume of this edition is entirely devoted to his *Researches on Nitrous Oxyde.*

Page 276. "In cutting one of the unlucky teeth called dentes sapientiæ, I experienced extensive inflammation of the gum, accompanied with *great pain*, which equally destroyed the power of repose and of consistent action." "On the day when the inflammation was most troublesome, I breathed three large doses of nitrous oxide. The pain always diminishes after the first four or five inspirations; the thrilling came on as usual," (in previous experiments,) "and uneasiness was for a few minutes swallowed up in pleasure."

Page 273. "Whenever its operation was continued to its highest extent, the pleasurable thrilling at its height about the middle of the experiment gradually diminishes; *the sense of pressure on the muscles was lost; impressions ceased to be perceived and voluntary power was altogether destroyed.*" Dr. Horace Wells of Connecticut was the first to apply it for the extraction of teeth; this was in 1844.

If nitrous oxide gas is inhaled for about thirty seconds and then taken away from the patient, violent muscular action attended with earnest declamation will, in the majority of cases, follow the removal of the inhaler, and these effects continue for about one minute, when the patient returns suddenly to his mental and physical equanimity.

When, however, this gas is inhaled for one and a half to two minutes, complete repose attended with perfect anæsthesia ensues. It is easy to administer the gas until complete anæsthesia is produced, for after the first three or four inspirations the patient eagerly desires more and the desire seems sympathetically continued even after the anæsthesia has resulted. The anæsthesia generally continues about 1½ to 2 minutes, and its influence upon the system about four or five minutes. The exciting effect is very similar to the operation of ether and chloreform when they, likewise, are administered in small doses, insufficient to produce anæsthesia. It will be seen therefore, that when a moderate quantity of this gas is used it acts as an exhilarant, producing intoxication; and when taken in large doses it induces narcotism and insensibility. By some it is thought to act as a stimulant; by others its action is considered similar to that of chloroform and ether, the retension of carbonic acid gas in the lungs. Fownes speaks of this gas as follows. "The most remarkable feature of this gas is its intoxicative power upon the animal system." "It may be respired, if quite pure, or merely mixed with atmospheric air, for a short time, without danger or inconvenience." "The effect is very transient, and is not followed by depression." Silliman says "It may be breathed without injury, but it should be quite pure and especially free from chlorine and inhaled through a wide tube." "The presence of chloride of ammonium in the nitrate employed should be especially avoided as producing chlorine." The same author also says that "in a few cases, dangerous consequences have followed its use, and it should always be employed with great caution." "In at least one case, in the laboratory of Yale College, it produced a joyous exhilaration of spirits, which continued for months, and permanent restoration of health." "Its effects, however, on different individuals, are various."

In certain conditions it may produce dangerous and even fatal results, but it is generally considered safer than Chloroform or Ether. We regard the indiscriminate use of nitrous oxide gas throughout the country at this time, without fatal results, as strong evidence of its safety as an anæsthetic agent; but at the same time would advise against its use where such disease exist as affections of the heart, active congestions or acute inflammation of the brain, lungs, or kidneys, or in a general plethoric condition, or where there is a tendency to hemorrhagic diathesis. In such cases as these its use as an anæsthetic agent is contraindictated.

(To be Continued.)

---

